# Food Diary Completion Does Not Significantly Impact Glycemic Control in an Observational Single-Institution Pilot Study of Patients with Type 2 Diabetes Mellitus

**DOI:** 10.7759/cureus.40596

**Published:** 2023-06-18

**Authors:** Amalie Alver, Kavita Batra, Arpita Basu, Kenneth Izuora

**Affiliations:** 1 Endocrinology, Kirk Kerkorian School of Medicine at the University of Nevada Las Vegas, Las Vegas, USA; 2 Emergency Medicine, University of Washington, Seattle, USA; 3 Research, Kirk Kerkorian School of Medicine at the University of Nevada Las Vegas, Las Vegas, USA; 4 Kinesiology and Nutrition Sciences, Kirk Kerkorian School of Medicine at the University of Nevada Las Vegas, Las Vegas, USA

**Keywords:** diabetes, food log, food and nutrition, food diary, diabetes type 2

## Abstract

Introduction: Hemoglobin A1c is frequently used to monitor glycemic control in patients with type 2 diabetes mellitus (T2DM). There is an association between dietary habits and hemoglobin A1c. Food diaries are often used to assist in dietary management and have been associated with modification of dietary habits. We aimed to investigate the impact of completing a seven-day food diary on hemoglobin A1c in subjects with T2DM. We hypothesized that patients who completed a food diary might independently modify their dietary habits, resulting in a clinically significant change in hemoglobin A1c.

Methods: Forty-five subjects with T2DM who completed a seven-day food diary were included in this observational study. Subjects had a mean hemoglobin A1c at baseline of 7.56 ± 1.43 and a body mass index of 33.6 ± 7.6 kg/m². A majority were female (57.8%) and insulin-dependent (71.1%). The primary outcome of interest was a clinically significant change in hemoglobin A1c (≥0.5%). Outcomes were assessed before and within six months after the food diary completion. Chi-square, independent-sample t-tests, and logistic regression were used to analyze the data.

Results: Twelve patients demonstrated a clinically significant decrease in hemoglobin A1c; however, this was not statistically significant regardless of gender (p = 0.5), baseline body mass index (p = 0.5), insulin use (p = 0.3), or community needs index (p = 0.7).

Conclusions: Although some patients had clinically significant improvements in their hemoglobin A1c, our findings suggest that the use of food diaries alone without goal-directed initiatives is insufficient to achieve differences in hemoglobin A1c in patients with T2DM and an average A1c within the range of 6%-8%.

## Introduction

Type 2 diabetes mellitus (T2DM) is one of the leading causes of morbidity and mortality in the world [1]. The number of patients living with T2DM globally has increased dramatically over the past 30 years and continues to trend upward [2]. Patients with T2DM are at a significantly increased risk for cardiovascular disease (CVD) and related complications compared to patients without diabetes [3,4]. Other well-known risk factors associated with increased CVD development include dyslipidemias and hypertension, which are often comorbidities that are also associated with T2DM. Optimal management of T2DM can be complex, requiring lifestyle modifications such as changes in dietary habits and levels of physical activity, education from qualified providers, various medication regimens, and coordination of patient care with specialists.

Monitoring hemoglobin A1c (HbA1c) allows for an estimation of the average blood glucose over a three-month period of time and is often used to evaluate glycemic control in patients with diabetes. In the National Health and Nutrition Examination Survey, elevated HbA1c levels were associated with increased mortality and CVD risk in type 2 diabetic patients [4]. A decrease of 0.5% or greater in HbA1c is considered clinically significant in patients with T2DM [5]. Patients with an HbA1c between 6% and 8% have been shown to have a significantly decreased risk of morbidity due to CVD compared to patients with values above this range [6]. Dietary habits have a substantial impact on patient health and are reflected in HbA1c values, lipid studies, and weight [7]. Monitoring the dietary habits of patients is commonly done by dieticians for education purposes, and food diaries are often used to assess diet quality [8,9]. Previous studies have indicated significant improvements in HbA1c when patients self-recorded their food intake with the specific intention of losing weight [10]. Patients who utilize food diaries for monitoring purposes may also, without explicit instruction, alter their eating habits independently due to the action of simply completing the food diary [11].

If patients self-modify their dietary intake by the act of completing a food diary, this may result in a clinically significant change in glycemic control and could have important implications for the management of patients with T2DM. To understand the impact of the completion of food diaries on glycemic control in patients with T2DM, a small cohort from a larger pool of subjects undergoing assessment of their dietary intake was enrolled in an observational substudy to determine if there were any significant differences before and after completion of a seven-day food diary. HbA1c was the main outcome of interest as a marker of glycemic control. Secondary outcomes investigated included changes in body mass index (BMI) as well as systolic and diastolic blood pressures (BP).

## Materials and methods

Eligibility criteria and data source

The primary study in which subjects were enrolled was a retrospective cross-sectional study at a university-based internal medicine clinic in Southern Nevada conducted from July 2019 through December 2021. Patients were eligible for enrollment if they were above 18 years and had been diagnosed with T2DM for at least one year. Subjects were required to complete a seven-day paper food diary over the course of the study. They were educated by trained clinical researchers on how to properly complete the food diaries, including methods for appropriately measuring their food and recording the times and locations of meals. Subjects were encouraged to include all food and beverages consumed over the course of the seven-day period in their food diary as detailed and accurate as possible. No specific education was given regarding glycemic control, weight loss, physical activity, or other modifiable lifestyle factors.

To be included in this substudy analysis, subjects must have completed one seven-day food diary fully. A chart review of the electronic medical record was conducted to obtain values of HbA1c, weight, BMI, and blood pressure before and after the completion of the seven-day food diary. The pre-study values obtained were the most recent data available before the start date of the food diary, and the post-study values were the most recent available after the completion of the food diary within a six-month time period from the date of food diary completion. Of the 71 potentially eligible subjects, 45 were included in this analysis. Subject identifiers were removed and entered into a database, and data analysis was conducted in a blind fashion.

Ethical considerations

The study was conducted according to the guidelines of the Declaration of Helsinki, and human subject review was approved by the Institutional Review Board of the University of Nevada Las Vegas (approved on 5/3/19). Written informed consent was obtained from all subjects involved in the study.

Variables and measures

The dependent variables of this study include change in HbA1c levels, change in systolic and diastolic BP, and change in BMI. We also re-categorized/dichotomized the change in HbA1c level as a clinically significant change in glycemic control categories (Yes versus No) using a threshold of -0.5% based on previous literature [5]. BMI categories were constructed as underweight, normal weight, overweight, and obese based on the Centers for Disease Control's (CDC) guidelines for practitioners [12]. We calculated the community need index (CNI) score for each participant. The CNI is a zip code-based score that accounts for a community’s unmet needs with respect to healthcare and is publicly accessible [13]. The CNI scores were re-categorized as low, mild, and high from the original categories of lowest: 1-1.7; second lowest: 1.8-2.5; Mild: 2.6-3.3; second highest: 3.4-4.1; and highest: 4.2-5.

Statistical methods

First, univariate and bivariate tests were conducted to analyze the data. All assumptions, including normality and homogeneity of variance, were assessed. Categorical variables were represented as frequencies and proportions, whereas continuous variables were represented by mean and standard deviations. The change in HbA1c (primary outcome) was used to indicate glycemic control, and the mean differences were calculated using a paired-sample t-test. The Chi-square/Fisher's exact test was used for comparing the categorical groups. Adjusted standardized residuals greater than 2 were considered significant cells for contingency tables larger than 2 × 2. A binomial logistic regression was performed to ascertain the effects of age, gender, CNI, baseline BMI and baseline blood pressure on the likelihood that participants have clinically significant glycemic control. The significance level was set at 0.05. The normal approximation to the binomial distribution method was used to calculate 95% confidence intervals of proportions in the univariate analyses. All analyses were conducted using SPSS version 28 (IBM Corp., Armonk, NY). We used a CHecklist for statistical Assessment of Medical Papers (CHAMP statement) for our results’ reporting [14].

## Results

Of the 71 subjects enrolled in the primary study, 45 (63.3%) subjects were included in this substudy analysis. About 26 subjects were excluded from this substudy analysis; 19 of those did not complete a seven-day food diary. The seven additional subjects who completed a seven-day food diary did not return to the clinic within six months of food diary completion and therefore did not have documented outcomes of interest in their electronic medical records. This resulted in 45 subjects that completed one seven-day food diary and had all data points of interest documented in their electronic medical records during a subsequent return visit within at least six months of food diary completion.

The sample was predominantly female (57.8%) and insulin-dependent (71.1%), with an average age of 64.9 ± 9.7 years. Subjects were stratified by pre-food diary BMI category, and at baseline, a majority had a BMI ≥ 30.0 (66.7%). A CNI of “high” was applicable to 77.8% of the study population; 13.3% were in the “mild” category, and only two subjects were found to have a low CNI (Table 1).

**Table 1:** Descriptive (univariate) statistics of 45 type 2 diabetes patients who completed a seven-day food diary ^a^BMI categories were constructed based on the CDC’s guidelines for practitioners [12]. ^b^CNI scores were re-categorized as low, mild, and high from the original categories of lowest: 1-1.7, second lowest: 1.8-2.5, mild: 2.6-3.3, second highest: 3.4-4.1, and highest: 4.2-5 [13]. ^c^A decrease in hemoglobin A1c of 0.5% or greater is considered clinically significant [5]. BMI: Body mass index; CNI: Community need index.

Variables	Characteristics	M ± SD	n (%)	95% confidence interval (lower, upper)
Age in years (mean ± standard deviation, [M ± SD])	-	64.87 ± 9.65	-	61.97, 67.77
Gender	Female		26 (57.8)	42.2, 72.3
	Male		19 (42.2)	27.6, 57.8
BMI categories (kg/m^2^)^a^	Normal (18.5-24.9)		8 (17.8)	8.0, 32.1
	Overweight (25.0-29.9)		7 (15.6)	6.5, 29.5
	Obese (≥30.0)		30 (66.7)	51.1, 80.0
Insulin-dependent	Yes		32 (71.1)	55.6, 83.6
	No		13 (28.9)	16.4, 44.3
Community need index^b^	Low (1-2.5)		2 (4.4)	0.5, 15.2
	Mild (2.6-3.3)		6 (13.3)	5.1, 26.8
	High (3.4-5)		35 (77.8)	62.9, 88.8
Clinically significant decrease in HbA1c (threshold: -0.5%)^c^	Yes		12 (26.7)	14.6, 41.9
	No		33 (73.3)	58.1, 85.4

Of these subjects, 12 subjects (26.7%) had a clinically significant decrease in HbA1c of greater than or equal to 0.5% over the course of the study with an equal number of males and females in this group (Table 1). A majority of these subjects who saw a decrease in HbA1c were classified as obese according to BMI (66.7%) and were also insulin-dependent (83.3%). None of the subjects who had a clinically significant change in HbA1c were found to have a low CNI score.

The average HbA1c at baseline for all subjects was 7.56 ± 1.43, which decreased slightly to 7.45 ± 1.44 over the course of the study (p = 0.4). Patients were then grouped to compare those that did have a clinically significant decrease in HbA1c with those who did not. Analysis of these two groups did not reveal any statistically significant difference to account for this change in HbA1c due to gender (p = 0.5), baseline BMI category (p = 0.5), insulin dependence (p = 0.3), or CNI (p = 0.7) (Table 2).

**Table 2:** Comparison of the characteristics of groups with or without a clinically significant change in glycemic control within six months of completing a seven-day food diary (N = 45)

Variables	Clinically significant change in glycemic control				P-values
	Yes, 12 (26.7%)		No, 33 (73.3%)		
Age in years (M ± SD)	63.75 ± 9.80		65.30 ± 9.71		0.6
Gender					
Male	6	50.0	13	39.4	0.5
Female	6	50.0	20	60.6	
BMI categories (kg/m^2^)					
Normal (18.5-24.9)	3	25.0	5	15.2	0.5
Overweight (25.0-29.9)	1	8.3	6	18.2	
Obese (≥30.0)	8	66.7	22	66.6	
Insulin-dependent					
Yes	10	83.3	22	66.7	0.3
No	2	16.7	11	33.3	
Community need index score					
Low (1-2.5)	0	0.0	2	6.1	0.7
Mild (2.6-3.3)	2	16.7	4	12.1	
High (3.4-5)	9	75.0	26	78.8	

Additional prepost comparison of primary and secondary outcomes for all subjects did not reveal any statistically significant change over the course of the study for HbA1c, BMI, or systolic and diastolic BP (Table 3).

**Table 3:** Prepost comparison of primary and secondary outcomes in a population of 45 subjects with type 2 diabetes mellitus who completed a seven-day food diary

Outcomes	Pre-intervention		Post-intervention		P-values
	Mean	SD	Mean	SD	
HbA1c %	7.56	1.43	7.45	1.44	0.4
Body mass index (kg/m^2^)	33.62	7.57	33.60	7.75	0.4
Systolic BP (mmHg)	130.64	16.67	131.02	15.29	0.8
Diastolic BP (mmHg)	75.62	8.80	75.76	9.18	0.8
Pulse pressure (mmHg)	55.02	13.09	55.27	13.46	0.9

The logistic regression model (Table 4) assessing the predictors of change in HbA1c explained only 12.3% (Nagelkerke R^2^) of the variance with a clinically significant change in glycemic control and correctly classified 79.1% of cases. Of the six predictor variables, no variables were statistically significant.

**Table 4:** Logistic regression predicting the likelihood of a clinically significant change in glycemic control based on selected independent variables in 45 type 2 diabetes mellitus patients within six months of completion of a seven-day food diary ^a^B: Unstandardized regression weight. ^b^SE: Unstandardized regression weight standard deviation. ^c^Wald: Test statistics. ^d^CI: Confidence interval.

Variables	B^a^	SE^b^	Wald^c^	P-values	Odds ratio	95% CI^d^ for Odds ratio (lower, upper)
Age	-0.043	0.041	1.110	0.292	0.958	0.884, 1.038
Gender (Reference: female)	0.918	0.851	1.165	0.280	2.504	0.473, 13.264
Baseline body mass index	-0.055	0.058	0.890	0.346	0.946	0.844, 1.061
Baseline systolic blood pressure	0.030	0.030	0.969	0.325	1.030	0.971, 1.093
Baseline diastolic blood pressure	0.016	0.057	0.080	0.777	1.016	0.908, 1.137
Community need index score	.065	0.551	0.014	0.906	1.067	0.362, 3.144
Constant	-2.312	5.026	0.212	0.646	0.099	-

## Discussion

Glycemic control in patients with T2DM is complicated. Medication regimens can be highly variable between patients [6]. Maintaining an HbA1c within the ideal range, which remains a reliable indicator of glycemic control, can be incredibly challenging. Diet quality has long been associated with improvements in HbA1c, with one longitudinal study of patients with type 1 diabetes who consumed higher levels of fiber and whole plant foods demonstrating better glycemic control [15]. Other findings report patients with T2DM benefit from consuming low-fat dairy and maintaining traditional diets such as the Mediterranean diet and the Korean diet [3,16]. Conversely, it has also been demonstrated that regardless of the type of food consumed (i.e., low-carbohydrate versus high-carbohydrate diets), patients with T2DM were able to achieve significant improvements in HbA1c and weight when they maintained a strict exercise regimen alongside a calorie-controlled diet [7].

The use of food logs has long been speculated to have an impact on eating habits [8]. A two-year lifestyle intervention program involving a food diary and exercise diary found that women were more likely than men to modify their dietary intake [17]. Subjects who had the most significant weight loss during this intervention also had the most significant improvement in glucose tolerance. However, there was also difficulty maintaining the improvement in glycemic control over a 21-month period. In another population of minority women with low socioeconomic status, subjects admitted to changing their eating habits when asked to log their meals [8]. They were also found to underestimate their total calorie intake by nearly 20% and substitute foods that were easier for them to record into their logs. Studies suggest that patients will alter their recorded food intake due to embarrassment or to avoid the inconvenience of completing the log in its entirety [18]. It is difficult to know if patients who underreport their caloric intake are purposefully altering their logs, rendering them inaccurate, or truly reducing their overall calories [19]. While these considerations are all important implications for the use of food diaries in diabetes management, this substudy analysis did not analyze food diary content; instead, it simply investigated the completion of the diary itself as an instigator for measurable change.

While there may not be a “one-size-fits-all” approach available, it is clear that dietary habits have a significant impact on health. Our findings suggest that in subjects with T2DM, completing a paper food diary without a specific goal-oriented intervention of glycemic control, such as increased nutrition education or additional lifestyle modifications, does not lead to clinically significant improvements in HbA1c, BMI, or blood pressure. These findings are consistent regardless of socioeconomic status, gender, age, or insulin dependence. Hildebrand et al. estimated that it takes nearly 24 hours of education with a patient to achieve an HbA1c decrease of 1%, and many patients with T2DM do not have access to this resource [20]. They also have found that less than 10% of patients receive diabetes education within their first year of diagnosis [20]. Patients who do not have appropriate education regarding the impact of their dietary habits on their diabetes may be less likely to realize the importance of their lifestyle choices [21].

It is also unclear if the specific type of food log used has an impact on patient modification of dietary habits. The use of interactive electronic diaries for insulin administration has shown improved glycemic control in some patients [9]. However, a study on diabetes control and nutrition outcomes in Australia did not find clear benefits to dietary consumption specifically when using an electronic food log [22]. The subjects in this study completed a paper food diary over the course of seven consecutive days. While this cohort was selected from a population of established clinic patients who may or may not have had prior nutrition education and counseling, no additional education or nutrition information about their dietary choices was provided over the course of diary completion.

Utilizing the paper food diary, 12 subjects did have a clinically significant decrease in their hemoglobin A1c; however, this was not found to be statistically significant. A similar pilot study sought to utilize an electronic diary for patients with T2DM to help manage their diets with a specific daily targeted total calorie intake goal over a six-month period [23]. Much like the results seen in our subject population, Inada et al. found that there was no significant change in subject HbA1c, total caloric intake, or body weight over the course of their study [23]. Their sample size was extremely limited (n = 7), and the average HbA1c in their population was nearly 7% at the beginning of data collection with exclusion criteria of >8.4%, which was similar to the findings of this study. Similar to that subject population, in this study with an average baseline HbA1c of 7.56%, subjects were already within the range of ideal glycemic control upon enrollment, which may have resulted in difficulty detecting a significant change in HbA1c [6]. Paper food diaries have some clear advantages over electronic food diaries as the cost of paper food diaries​​​​​​​ is significantly lower, and it is simple and easy to hand a paper log to a patient in the clinic and review it at subsequent visits. This is especially relevant for older patients with diabetes who may not be skilled in using technology or have other barriers ​​​​​​​to using small electronic devices, such as vision impairment.

This study has limitations. Overall, subject recruitment was significantly disrupted by the COVID-19 pandemic, and many subjects who enrolled in the primary study did not complete food diaries as anticipated. In addition, several subjects who did complete the food diaries did not return to the clinic in person during the COVID-19 pandemic and therefore did not have outcomes of interest documented in their electronic health records, leading to their exclusion from the substudy analysis and ultimately a significantly smaller sample size than anticipated. Moreover, this study was based on the data from a single institution, which may impact the external validity and thus the generalizability of the study. While the clinically significant decrease in HbA1c achieved by some patients is promising, a larger sample size may be helpful in detecting any statistically significant change that occurs.

## Conclusions

In conclusion, our findings suggest that the use of paper food diaries alone without goal-directed initiatives and nutrition education is not sufficient to achieve significant differences in HbA1c in patients with type 2 diabetes mellitus who have an average A1c value within the ideal range of 6%-8%. The authors recommend larger sample sizes and subjects with broader HbA1c ranges be included in future studies to further explore clinically relevant changes that could result from the process of completing a food diary. As a small number of our subjects did have a clinically significant decrease in their hemoglobin A1c, primary care providers may find it in their interest to incorporate a paper food diary into their practice as it may benefit a small number of their patients. Practitioners could utilize this food diary to review at visits to encourage patients in lifestyle modifications and reinforce healthy food habits, much like a blood pressure log. This may be especially helpful for patients who do not have access to registered dieticians or nutritionists. Future research should also consider investigating the use of paper versus electronic food diaries to determine if there is a superior method of documenting dietary habits with the intention of improving glycemic control.
